# Transient Thermal Tensile Behaviour of Novel Pitch-Based Ultra-High Modulus CFRP Tendons

**DOI:** 10.3390/polym8120446

**Published:** 2016-12-21

**Authors:** Giovanni Pietro Terrasi, Emma R. E. McIntyre, Luke A. Bisby, Tobias D. Lämmlein, Pietro Lura

**Affiliations:** 1 Mechanical Systems Engineering Laboratory, Empa, Swiss Federal Laboratories for Materials Science and Technology, Überlandstrasse 129, CH-8600 Dübendorf, Switzerland; Tobias.Laemmlein@empa.ch; 2 BRE Centre for Fire Safety Engineering, Institute for Infrastructure & Environment, School of Engineering, University of Edinburgh, The King’s Buildings Edinburgh, EH93JL Scotland, UK; s0679623@sms.ed.ac.uk (E.R.E.M.); Luke.Bisby@ed.ac.uk (L.A.B.); 3 Concrete and Construction Chemistry Laboratory, Empa, Swiss Federal Laboratories for Materials Science and Technology, Überlandstrasse 129, CH-8600 Dübendorf, Switzerland; pietro.lura@empa.ch; 4 Institute for Building Materials (IfB), ETH Zurich, 8092 Zurich, Switzerland

**Keywords:** carbon fibre reinforced polymers (CFRPs), high elastic modulus, tensile properties, high temperature behaviour, dynamic mechanical thermal analysis (DMTA), thermogravimetric analysis (TGA)

## Abstract

A novel ultra-high modulus carbon fibre reinforced polymer (CFRP) prestressing tendon made from coal tar pitch-based carbon fibres was characterized in terms of high temperature tensile strength (up to 570 °C) with a series of transient thermal and steady state temperature tensile tests. Digital image correlation was used to capture the high temperature strain development during thermal and mechanical loading. Complementary thermogravimetric (TGA) and dynamic mechanical thermal (DMTA) experiments were performed on the tendons to elucidate their high temperature thermal and mechanical behaviour. The novel CFRP tendons investigated in the present study showed an ambient temperature design tensile strength of 1400 MPa. Their failure temperature at a sustained prestress level of 50% of the design tensile strength was 409 °C, which is higher than the failure temperature of most fibre reinforced polymer rebars used in civil engineering applications at similar utilisation levels. This high-temperature tensile strength shows that there is potential to use the novel high modulus CFRP tendons in CFRP pretensioned concrete elements for building applications that fulfill the fire resistance criteria typically applied within the construction industry.

## 1. Introduction

In the last decade, the precast concrete industry has been progressively developing a range of advanced thin-walled concrete elements utilizing high-performance, self-consolidating concrete reinforced (or prestressed) with high-strength, lightweight, and non-corroding carbon fibre reinforced polymer (CFRP) grids or tendons [1,2,3,4]. Due in particular to the stress corrosion resistance of CFRP reinforcements, the concrete cover is not determined by corrosion protection or carbonation issues, as is the case for steel reinforcement and prestressing, and the concrete cover in these bending elements can therefore be reduced to a minimum (e.g., 10–20 mm), leading to lighter and more durable precast elements. The concrete cover size is now determined by static considerations (reception of the compressive stresses due to bending and of the splitting tensile stresses in the prestress transfer zone of prestressing tendons at the beam ends) and by the mismatch in thermal expansion coefficient between CFRP (transverse to fibre direction [1]) and high-performance concrete [5]. One example of this trend is a new type of precast CFRP pretensioned concrete element intended as load-bearing panels for glass/concrete building facades [1].

Adequate load carrying capacity during exposure to fire is crucial for applications of CFRP precast concrete structural elements in both the interior and exterior of buildings. Adequate fire resistance is a legitimate concern for these novel precast CFRP reinforced high strength concrete elements, since it is known that the tensile strength of CFRP and the bond strength between CFRP reinforcing tendons and concrete deteriorate comparatively quickly at elevated temperature [6,7]. It is also widely known that high-strength concrete show an increased propensity for heat-induced explosive spalling failure when subjected to fire [8]. The tensile and bond strength decreases experienced in a fire situation, and their impacts on the load-carrying capacity of reinforced and prestressed concrete structures, remain largely unknown particularly for prestressing applications. The spalling behaviour of high-strength concrete can be effectively mitigated by advanced concrete mix design incorporating a suitable quantity and type of polypropylene microfibre [9].

This paper provides insights into the high temperature thermal and tensile strength behavior of novel UHM CFRP tendons that have the potential to effectively reinforce/prestress high-strength concrete elements. The high modulus of the carbon fibers in particular allows considerable load transfer to the fibers in concrete at low strains of the composite (assuming an adequate and durable bond), making these materials suited for reinforcement and prestressing applications in durable, thin-walled concrete precast elements.

A tensile testing machine equipped with a thermal chamber was used to test the novel prestressing tendons under both transient and steady-state high temperature conditions (between 300 and 570 °C). Digital image correlation (DIC) enabled the assessment of strains in the CFRP during elevated temperature testing. The aim of these experiments was to characterize the high-temperature tensile response of a specific, novel ultra-high modulus (UHM) pitch-based carbon fibre reinforced polymer (CFRP) tendon and to compare the observed behaviour against that of conventional CFRP prestressing tendons based on polyacrylonitrile (PAN) carbon fibres, as well as to conventional prestressing steel wires. Additional thermo-mechanical analysis (DMTA) and thermogravimetric (TGA) experiments were undertaken to shed further light on the high temperature tensile strength behaviour of the novel reinforcing bars.

## 2. Materials Tested

New pitch-based high modulus carbon fibres with an attractive performance/price ratio are increasingly available for the construction materials market. Examples of such fibres are given in [9], describing industrial-grade fibre rovings composed of 12,000–16,000 single carbon filaments, with an elastic modulus of 640–790 GPa and a tensile strength varying between 2600 and 3200 MPa. Their cost is approximately 50 Euro/kg, which is twice the cost for conventional high strength carbon fibres. Considering that the cost of a carbon fibre reinforced polymer (CFRP) tendon with diameter 5.4 mm is approximately 4 Euro/m when using conventional high strength carbon fibres, we can estimate the price impact of the new high modulus fibres. If we assume that the novel UHM tendons can be produced by pultrusion, the resulting pultruded tendon price would increase approximately to 4.7 Euro/m, which is a 17% increase in price (at a 20%–30% lower tensile capacity). The high tensile strength, corrosion resistance, and low density (2.15 g/cm^3^ [10]) of these fibres make them attractive for structural engineering applications. The high modulus of the fibres in particular allows considerable load transfer to the carbon fibres in hybrid material systems at low strains/deformations of the composite (assuming an adequate and durable bond). The drawback of the pitch-based fibres is a comparatively low failure strain for the novel fibres and therefore for tendons manufactured from these fibres; this is between 0.3% and 0.4% [10] and must be carefully considered in design of an FRP prestressed structure when using these materials. It is noteworthy that prestressing steel has drastically different tensile behaviour, and is typically considered an ideally elastic-plastic reinforcement in design, with a plastic limit strain of between 0.1% and 0.2%.

**Figure 1:**
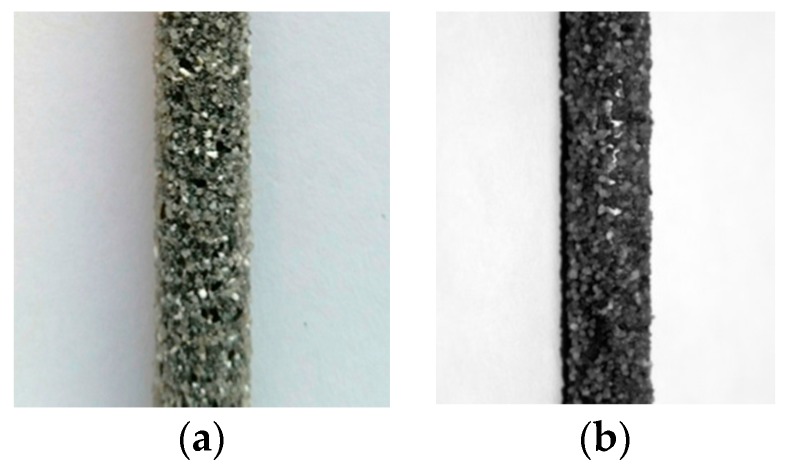
(**a**) Novel sand coated ultra-high modulus (UHM) pitch-based carbon fibre reinforced polymer (CFRP) and (**b**) conventional polyacrylonitrile (PAN)-based CFRP tendon.

The study presented in this paper was focused on a novel type of CFRP tendon, for possible use in civil engineering concrete prestressing applications [11], composed of 66% by volume Mitsubishi DIALEAD™ K13916 fibres [9]. The novel CFRP prestressing tendons have a diameter of 5.3 mm (with a standard deviation of ±0.1 mm) and were produced batch wise by a tape-laying method [12] at a total length of 3.2 m per tendon. To accomplish this, unidirectional pre-impregnated (prepreg) layers were laid between two pins and then wrapped in the hoop direction with a carbon fibre roving to press them transversely and form a cylindrical geometry. The commercial preimpregnated fibre rovings used had an epoxy polymer matrix (huntsman XB3515/AD5021 [13]), and were hardened for 1.5 h at 100 °C followed by 2 h at 140 °C before demolding. The tendons’ surface was subsequently coated with a lamination epoxy (araldite LY 5052 [13]), and, to promote a strong bond with the surrounding concrete, the tendons were sand coated by broad-casting silica sand (with a mean diameter of 0.5 mm). The coating was hardened for two additional hours at 125 °C. The resulting novel UHM CFRP prestressing tendons are shown in Figure 1a.

**Figure 2:**
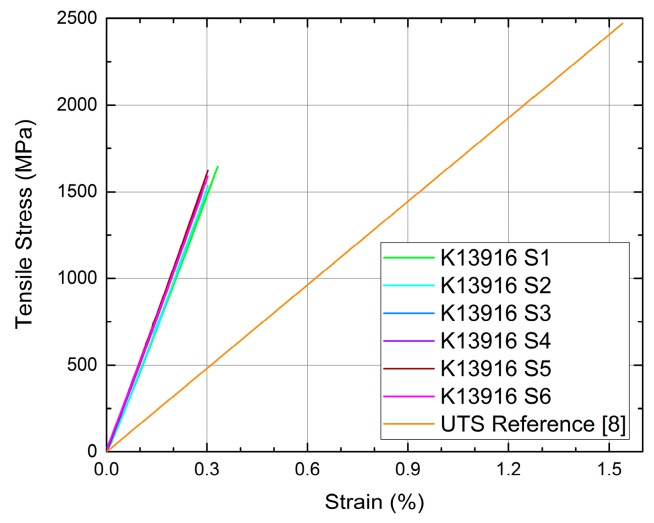
Tensile stress versus strain response of new UHM CFRP tendons (6 specimens labelled K13916 S1 to S6), compared with a conventional CFRP prestressing tendon [8].

These tendons behave linear elastically until tensile failure (Figure 2), have an average tensile strength of 1561 MPa (with a standard deviation of ±68.05 MPa from six tensile tests) and an average *E*_11_ modulus (measured following [14]) of 509 GPa (±13.49 GPa). The resulting Design Tensile Strength (DTS) is 1400 MPa. The DTS was calculated from the results of the six tensile tests shown in Figure 2 using the average measured strength of 1561 MPa (Table 1), the standard deviation of 68.05 MPa and assuming a normal distribution of the tensile strengths (which is one possible assumption following [15]). The DTS was defined as the average tensile strength minus 2.3 standard deviations, corresponding to the 2.1 percentile.

**Figure 3:**
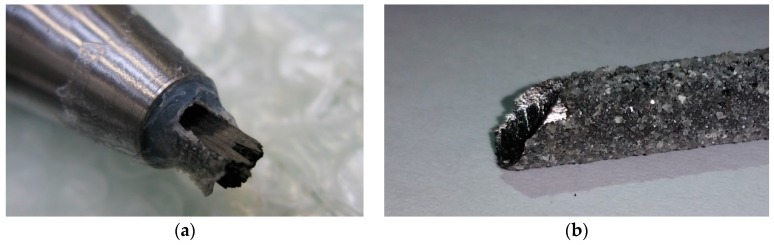
(**a**,**b**) Brittle failures of two UHM CFRP tendons at the load entrance of the resin-cast metallic barrel anchor used to anchor the tendons during the tensile tests.

Figure 3a shows the brittle failure that was observed during tensile testing of the CFRP tendons at the loaded end of the resin-cast anchorage that was used to grip the tendon during testing. A close-up of the brittle CFRP failure surface at the same location is given in Figure 3b. This failure mode, and the resulting tensile capacity of the tendons, was clearly influenced by the complex stress state at the loaded end of the resin cast anchorage system [11]. A combination of tensile stress along the tendon axis, a superimposed compression (clamping) stress transverse to the tendon axis, and a peak in shear stress at the entrance of the tendon to the anchorage caused a premature and brittle shearing-off the CFRP at this location. A potential solution to the pronounced strength anisotropy and matrix-bond problem of the UHM fibres [15] would be to design a tensile anchorage system and corresponding test setup which leads to smaller transverse stress peaks. Analysis of bending tests of under-reinforced concrete beams prestressed with these UHM tendons (Empa, unpublished results) showed that when designing for the tensile failure of the CFRP tendon, tensile stresses in the outermost tendon reached levels around 1900 MPa at flexural failure of the beam.

According to the rule of mixtures for unidirectional fibre reinforced polymers [15], and considering that the Mitsubishi DIALEAD™ K13916 carbon fibres have a guaranteed tensile strength of 3200 MPa [10], the theoretical tensile strength of the UHM CFRP tendons is 0.66 × 3200 MPa = 2112 MPa, which is 35% higher than the average tensile strength measured in the tests described above. This is likely due to the observed premature failure mode next to the anchors (see Table 1). A similar approach shows that the experimentally-determined tensile elastic (*E*_11_) modulus of the novel tendons (509 GPa) is accurately estimated with the rule of mixtures [15], which suggests to 0.66 × 760 GPa = 502 GPa.

The thermomechanical behaviour of the new UHM tendons was compared against conventional CFRP tendons used for concrete pre-tensioning applications [1,6,11]. The more conventional CFRP tendons have a nominal diameter of 5.4 mm (±0.1 mm) and were produced by pultrusion. PAN based carbon fibres in the conventional tendons were TENAX™ UTS 5631 [16] at a carbon fibre volume fraction of 65%. These tendons had an epoxy polymer resin matrix consisting of Bakelite Ruetapox VE 4434 resin [17]. A sand coating was also applied to the surface of these bars by in-line spray-coating silica sand onto a coating of epoxy resin (araldite LY 5052 [13]), applied after the primary pultrusion and curing process, as described previously, and resulting in a coating approximately 0.5 mm thick. The CFRP tendons’ average nominal tensile strength was 2471 MPa (standard deviation of ±168 MPa from 45 tensile tests), with an elastic modulus *E*_11_ [14] of 156 GPa. The tensile stress-strain relationship for the reference CFRP tendons was linear-elastic to failure (Figure 2), and displayed an average ultimate strain of 1.54% [1]. Both CFRP tendons studied herein have a nominal mass of 0.123 kg/m, which is about half the mass per unit length of a standard 6 mm diameter cold-drawn steel prestressing wire [18], and about 47% of the mass per unit force. A sample of the reference CFRP prestressing tendon is shown in Figure 1b.

## 3. Experimental Programme

The primary aim of the study presented herein was to experimentally characterize the transient elevated temperature tensile behaviour of the novel pitch-based carbon fibre reinforced epoxy tendons described in the previous section. It was of interest to compare the response of the novel tendons with the behaviour [6] of the conventional normal modulus CFRP prestressing tendons based on Polyacrylonitrile (PAN) carbon fibres. Two sets of high-temperature experiments were performed for the new UHM tendons in a materials testing frame equipped with a high temperature environmental chamber. The experiments consisted of 11 transient elevated temperature tensile tests—In which the CFRP was loaded to a sustained load and then heated under constant load until failure—Followed by two steady state high temperature tensile tests of the new UHM tendons at 300 °C, in which the tendons were heated to a constant temperature and then loaded under constant temperature until failure.

**Figure 4:**
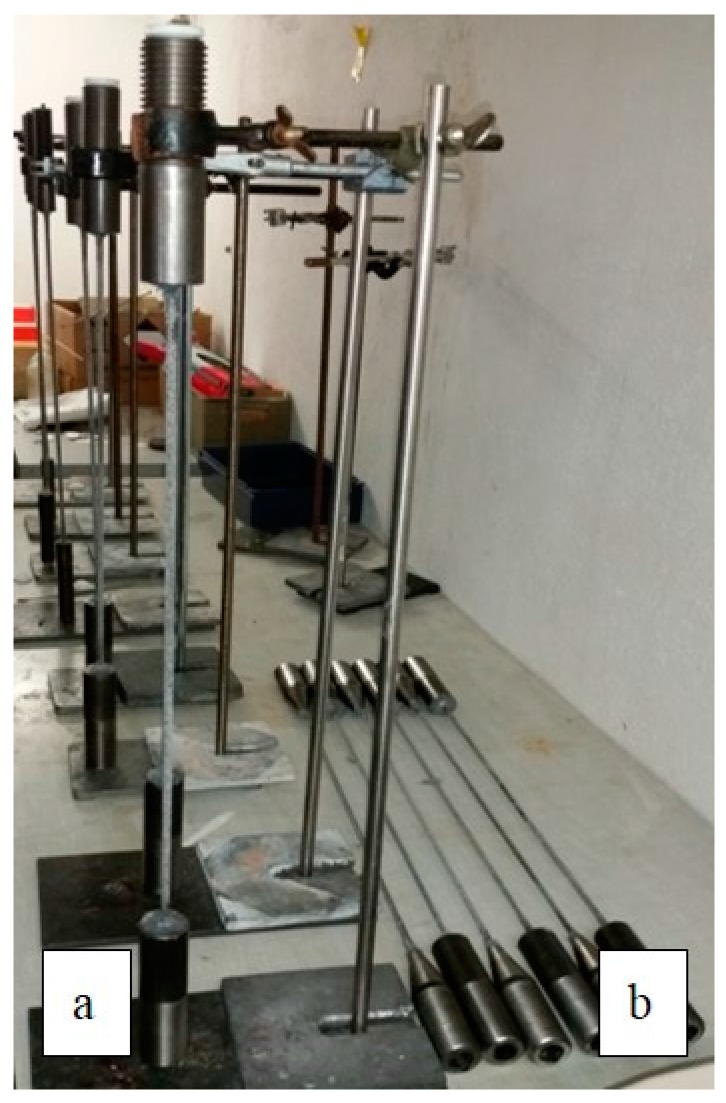
(**a**) Setup to cast epoxy grout anchorages; (**b**) Six CFRP specimens with testing anchors installed.

**Figure 5:**
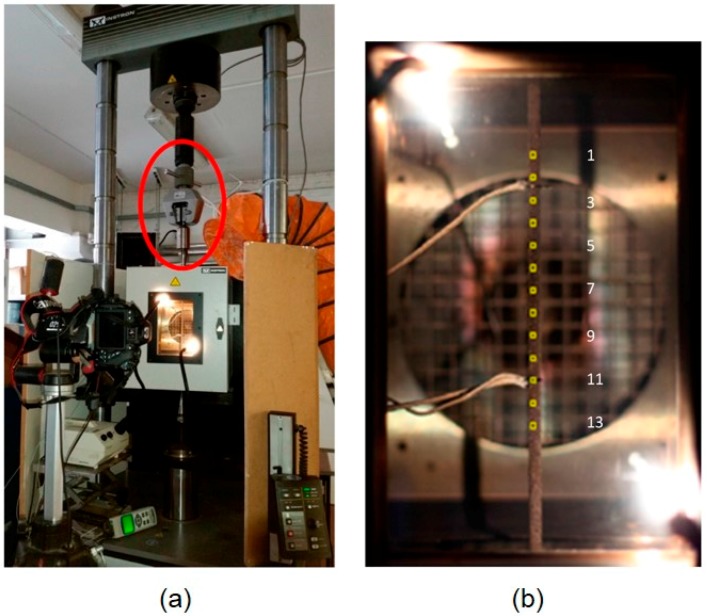
(**a**) Test setup with DIC camera in front of Instron 600 LX tensile testing machine equipped with environmental chamber; the upper tendon’s anchorage clamping device is highlighted; (**b**) Detail showing a CFRP specimen with three attached thermocouples and the DIC patch alignment/numbering for strain measurement.

The total length of each tensile specimen was 810 mm, which led to a free testing length of 500 mm between the 140 mm long steel barrel anchorages (Figure 4). The crucial issue of gripping the tendons, which is far from straightforward for this type of tendon, was addressed by epoxy bonding high strength steel sockets (Grade ETG 100 [19]) to both tendon ends. After spraying a polytetrafluoroethylene (PTFE) separating layer on the anchorages’ inner surfaces, the CFRP tendon specimens were centered and fixed with a silicone drop at the load entrance of the steel anchorages. A low-viscosity lamination epoxy resin (Araldite LY 5052 [13]) was then cast into the conical interior of the cylindrical steel sockets [5]. Half of the steel anchorages were ground to a conical shape at the load entrance (Figure 3a and Figure 4) in order to make this region of the socket softer, which gave better results when performing tensile tests at ambient temperature [5]. An M36 ISO metric screw thread was cut into the external surface of the steel anchorages in order to fix them to steel adaptors that were then clamped into the wedge action grips of the materials testing frame, as shown in the highlighted area of Figure 5a.

A high-capacity materials testing frame at the University of Edinburgh’s BRE Centre for Fire Safety Engineering (Edinburgh, Scotland, UK), which includes an in-line environmental chamber, was used for the tensile tests (Figure 5). The setup consisted of an Instron 600 LX static hydraulic universal testing machine (Instron, Norwood, MA, USA) with a tension/compression load capacity of 600 kN, equipped with an Instron CP103790 high temperature environmental chamber (Instron, Norwood, MA, USA) capable of covering a temperature range from −70 to +620 °C (temperature stability ±2 °C, heating rate 10 °C/min). The anchorages of the tensile specimens were clamped using spherically seated wedge action grips with 150 kN capacity both above and below the environmental chamber.

The transient thermal tensile tests were performed at a crosshead stroke rate of 0.5 mm/min up to the predefined prestress level, and three representative prestress levels were studied; namely 40%, 50%, and 60% of the tendon’s design Ultimate Tensile Strength of 1400 MPa. These prestress levels are representative of the long-term in service stresses expected for CFRP tendons in pre-tensioned precast concrete elements [1] in practice. The CFRP tendons were thus stressed to corresponding service stresses of 560, 700, and 840 MPa, with three or four specimens tested at each stress level, and then heated at a rate of 10 °C/min under a sustained load control mode until failure (or until the maximum stable temperature of 570 ± 2 °C of the environmental chamber was reached).

For the two steady state tests, the UHM specimens were prestressed to a small stress of only 100 MPa during heating up to 300 °C, again at a heating rate of 10 °C/min, and then loaded to failure under a crosshead stroke controlled loading at a rate of 0.5 mm/min.

One K-type ThermoCouple (TC) (denoted as TC_2_, Empa, Dübendorf, Switzerland) was positioned in the gas phase at the center of the testing chamber, and was used to control the chamber’s temperature. Three additional type K thermocouples (denoted as TC_1_, TC_3_, and TC_4_) were tied onto the CFRP specimen using 0.3 mm steel wires: TC_1_ at 95 mm and TC_3_/TC_4_ at 190 mm from the top inner surface of the temperature chamber (see Figure 5). These additional TCs were used to monitor the temperature of the tendon and the uniformity of temperature within the chamber and on the test specimens.

A digital single lens reflex (DSLR) camera of type Canon 650d (18 Megapixel CMOS, 50mm fixed focal length lens, Canon Inc., Tokyo, Japan) was used for DIC analysis to measure the axial deformations of the tendons during the tensile tests (see Figure 5). The camera was used to acquire high-resolution digital images of the sand-coated surface of the CFRP tendons, at a rate of 0.2 Hz. A custom-coded Matlab module called GeoPIV (Version RG, Queen’s University, Kingston, ON, Canada) was used for the DIC analysis; this was developed by Take et al. at Cambridge University, UK, and Queen’s University, Canada [20]. The initial DIC analysis was carried out with 32 × 32 pixel patches (~2.3 mm square in the images based on an average scale factor amongst the tests). Data from this analysis displayed considerable scatter, so an additional DIC analysis was performed using 64 × 64 pixel patches, which significantly improved the output. Thirteen patches were used in a vertical formation on each CFRP bar, as shown in Figure 5b. The initial stress vs. DIC-derived strain behaviour showed an increase in stiffness during the loading phase, before starting the transient tensile thermal experiments. This indicated minor out-of-plane movement of the test specimen in the early stages of loading (i.e., during seating of the specimens). Linear extrapolation, using a tangent determined in the tensile stress range between 300 and 700 MPa, was used to correct for this error.

Thermogravimetric analysis (TGA) (A Netzsch TG 209 F1, Netzsch GmbH, Selb, Germany) was performed on the UHM tendon material to characterise its thermal degradation in an unstressed state. Two uncoated samples of the CFRP tendon core material (discs of 3 mm diameter and approximately 1 mm height) were cut with a diamond saw from a tendon that had previously had its sand coating mechanically removed. The two samples were cleaned with acetone , had starting weights of 12.2 mg, and were conditioned in a storage room at 23 °C and 50% Relative Humidity (RH) before performing the TGA tests. A Netzsch TG 209 F1 Iris thermos-microbalance was used, with tests performed in air at a heating rate of 20 °C/min over the range from 25 to 900 °C. The testing precision was ±0.01 mg.

Dynamic Mechanical Thermal Analysis (DMTA) was also performed on two specimens of the novel UHM tendons, to provide further insights into the thermomechanical response of the novel material and, crucially, to determine its glass transition temperature (*T_g_*) by various possible definitions. A dynamic mechanical thermal analyser EPLEXOR 500 (Gabo Qualimeter GmbH, Ahlden (Aller), Germany), with built-in displacement sensors and a 500 N load cell, was used. For each 50 mm long specimen, two DMTA tests were performed in a 3-point bending mode in accordance with [21] (displacement controlled, 40 mm span). The static base load was chosen to induce a 0.2% tensile strain at mid-span, over which a dynamic displacement causing ±0.082% strain amplitude was superimposed at a frequency of 1 Hz. The temperature range was between 20 and 210 °C, at a heating rate of 2 °C/min. The sand coating was removed from the original tendons at their support locations and the specimens were stored in lab conditions before testing (1 month in a 23 °C/50% RH climate). The *T_g_* values were calculated both using an *E’* (storage modulus) onset definition, and using a peak tan δ definition [21].

## 4. Results

Selected results of the transient thermal tensile tests are summarized in Table 2. At 50% of DTS the average failure temperature of the new UHM tendons was 409 °C (from four repeat tests with a standard deviation of ±8.3 °C), while at 60% of DTS the average failure temperature decreased to 395 °C (three tests, ±9 °C). The failure temperature in these tests was defined as the average temperature of the measuring thermocouples attached to the tendon, at the moment when loss of prestress occurred; this was initiated by the successive tensile failure of the near-surface fibres (which were at this point ‘dry’ due to the epoxy matrix having pyrolysed). For the lowest prestress level of 560 MPa (corresponding to 40% of DTS), failure was not reached during any of the tests. Thus, after holding the load constant for 10 min at a temperature of 570 °C, it was decided to increase the tensile stress from 560 MPa up to failure (at a crosshead displacement rate of 1 mm/min) to determine the remaining tensile strength at that temperature. The resulting average remaining tensile strength was 644 MPa (±18 MPa); this corresponds to 46% of DTS.

In the two steady-state tensile tests, the UHM specimens were prestressed to 100 MPa and then heated to 300 ± 5 °C. A crosshead displacement controlled loading was then applied until failure. The first specimen failed at 1004 MPa (72% of DTS), whereas the second failed at 1037 MPa (74% of DTS).

**Figure 6:**
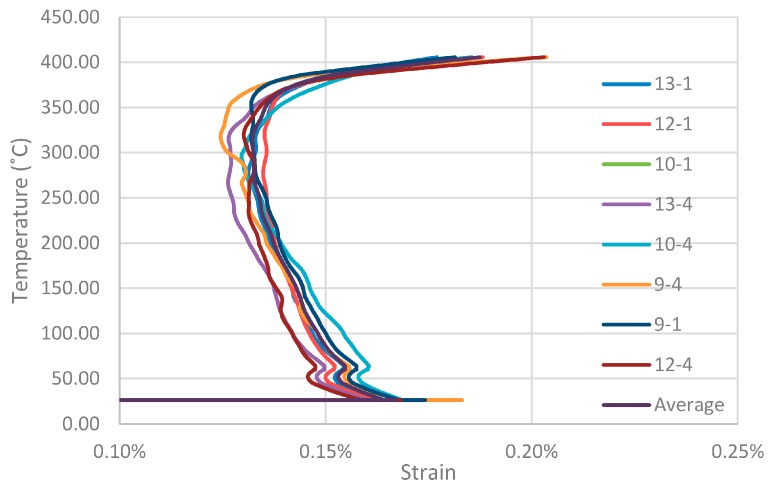
DIC-measured tensile strain versus temperature curves for the transient thermal tests of Specimen 50c subjected to a prestress of 700 MPa (50% of DTS).

**Figure 7:**
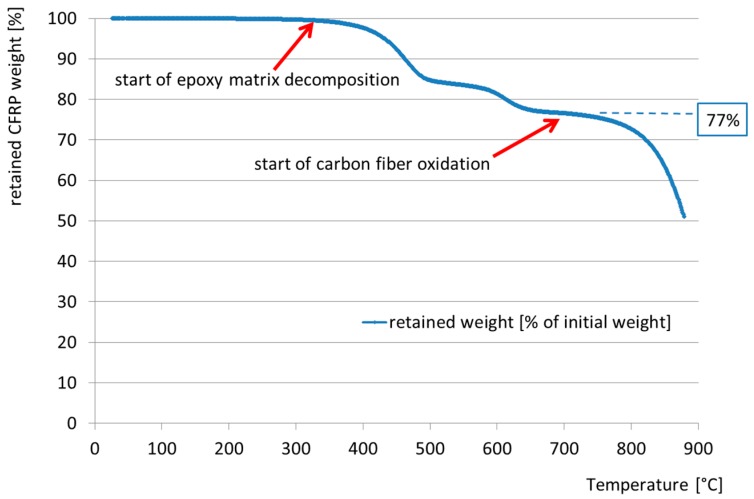
Thermogravimetric (TGA) plot (mass loss vs. temperature) for the UHM CFRP material.

Figure 6 shows typical tensile strain versus temperature curves for one of the specimens loaded at 50% of DTS (Specimen 50c), with the strains captured using DIC by measuring the increase in spacing between two patches (e.g., ‘13-1’ denotes the average strain measured between patches no. 13 and no. 1 in Figure 5b). It is noteworthy that these data are smoothed based on a LOcal regression (LOESS) model [22]. The longitudinal tendon strain increases linearly during the initial loading phase to reach the predefined prestress level corresponding to the initial horizontal plateau in the data in Figure 6. Thereafter, the temperature is increased at a rate of 10 °C/min until failure, and the resulting strains are evident. Given that the fibres in the UHM specimens are carbon, it was expected that there would be a small strain reduction during the heating phase of the experiment (since the bars’ coefficient of thermal expansion in the fibre longitudinal direction CTE_11_ is slightly negative, i.e., −1.2 × 10^−6^ in the range between 50 and 125 °C [10]). This expectation is confirmed by the data up to temperatures of approximately 250 °C. Figure 6 clearly shows a turning point in the data in the temperature range between 270 and 320 °C, where the strains begin to increase on further heating. For all specimens prestressed at 50% of DTS and investigated with DIC during testing, this temperature range was determined to be between 260 and 360 °C based on the minimum strain value observed after the onset of heating. Based on the TGA data (see Figure 7), this range indicates the onset of matrix thermal decomposition. It is noteworthy that the TC readings during testing are surface temperatures, and do not necessarily represent the tendon core temperature. The specimen failure was signaled by rapidly increasing strains starting at approximately 360 °C, somewhat below the average failure temperatures reported in Table 2. The decomposition of the epoxy from the bars’ surface inwards, combined with creep of the epoxy matrix inside the bars, may explain this difference.

The strain at failure of the tendons (0.18%–0.22% for a sustained stress of 700 MPa, as shown in Table 2) has been reduced based on the expected quasi-static strain at 700 MPa to filter out the bar movement during the loading phase. On this basis, the strain calculations assume that the distance between the camera lens and the foremost surface of the bar is unchanged from the initial calibration carried out at the start of the experiment; therefore it is expected that the results will contain some small calibration errors, and as such should only be considered as an illustrative estimate.

**Figure 8:**
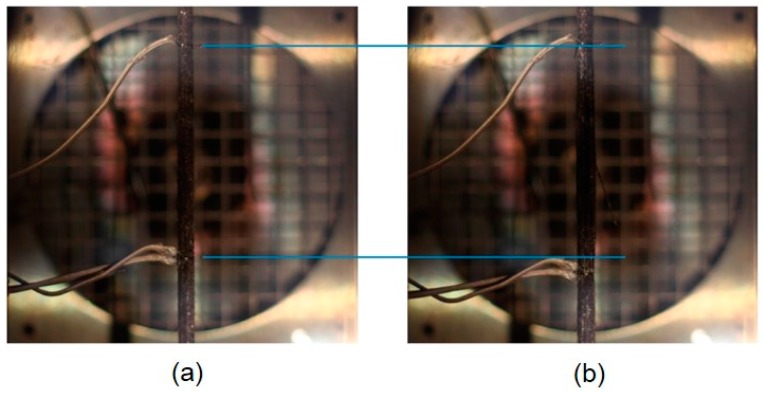
CFRP specimen prestressed at 700 MPa (Specimen 50d) immediately before (**a**) and after failure (**b**).

Figure 8 shows one UHM CFRP specimen prestressed at 700 MPa (50% of DTS) immediately before and after failure (at 410 °C in this case). At temperatures above 400 °C, the epoxy matrix is completely decomposed and the ‘dry fibres’ are carrying the applied loads (albeit with some charred matrix residue remaining). The fibres relax after failure and the cross section physically opens (Figure 8 right). The sudden increase in strain is clearly visible comparing these two images using the auxiliary horizontal blue lines drawn.

The relative retained mass versus temperature measured for the novel UHM-CFRP in the TGA is given in Figure 7. The thermal degradation of the epoxy resin (which makes up 23% of its initial mass) starts at approximately 300 °C and ends at approximately 680 °C. The authors hypothesize that the first mass drop represents the pyrolysis of the epoxy matrix, and the second (starting at approximately 570 °C) is caused by char/residue oxidation, while the oxidation of the carbon fibres themselves (the remaining 77% of the mass of the tendon) follows from about 680 °C [23], and continues until 900 °C (the end of the TGA test).

**Figure 9:**
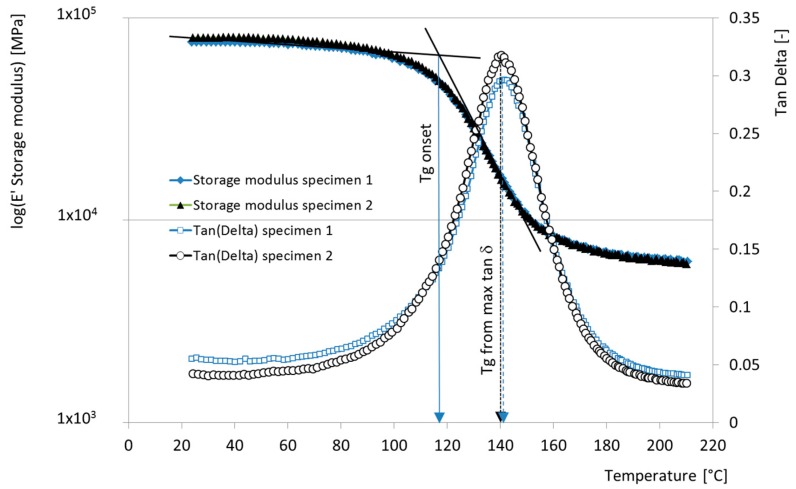
Results of DTMA analysis, *E’* and tan δ versus temperature for the novel UHM CFRP tendons.

The DMTA experiments of two specimens of the novel UHM tendons gave more insight into their thermo-mechanical response (Figure 9). The storage modulus *E’* (primary axis) versus temperature is depicted next to the tangent of the phase shift angle δ (on the secondary axis) versus temperature. The two specimens were measured in two consecutive DMTA heating runs between 20 and 210 °C. The results of the second heating run produced essentially the same *E’* versus temperature and tan δ vs. temperature curves as in the first run, and are not reproduced in Figure 9.

Table 3 summarizes the *T_g_* values determined following a ‘Peak tan δ’ definition [21] and a more conservative ‘*T_g_* onset’ definition (used in [6]). As already noted, each sample was retested with the same DMTA protocol to observe whether an increase in *T_g_* would take place. This would be a clear sign of a post-hardening of the epoxy matrix of the tendon during the first heating cycle. No such shift in *T_g_* was observed, and the *T_g_* values measured for the second DMTA run were between 0.9 °C and 2.3 °C lower than in the first measurement run (see Table 3); this is within the uncertainty of the instrumentation used (±3 °C). The DMTA results for the novel UHM tendons are similar to those obtained in [1,5] for the conventional CFRP tendons (properties in Table 1 and further elaborated in [1]). The average *T_g_* onset for the conventional tendons was observed to be 121 °C [6], and the average *T_g_* (peak tan δ) was 135 °C [1].

## 5. Discussion

The high-temperature tensile strength results of the novel UHM CFRP tendons (Table 2) are considered to be comparatively good when compared to the high temperature performance of typical FRP reinforcements for concrete structures. A literature review in [7] concluded that, at temperatures between 250 and 400 °C, most carbon/epoxy CFRP composites lose about half of their original tensile strength. The novel UHM CFRP tendons investigated in the present study have a design tensile strength of 1400 MPa, and their failure temperature at a prestress of 50% of the design tensile strength was found to be 409 °C (Table 2), which is at (or above) the upper end of the accepted temperature range for this stress value.

**Figure 10:**
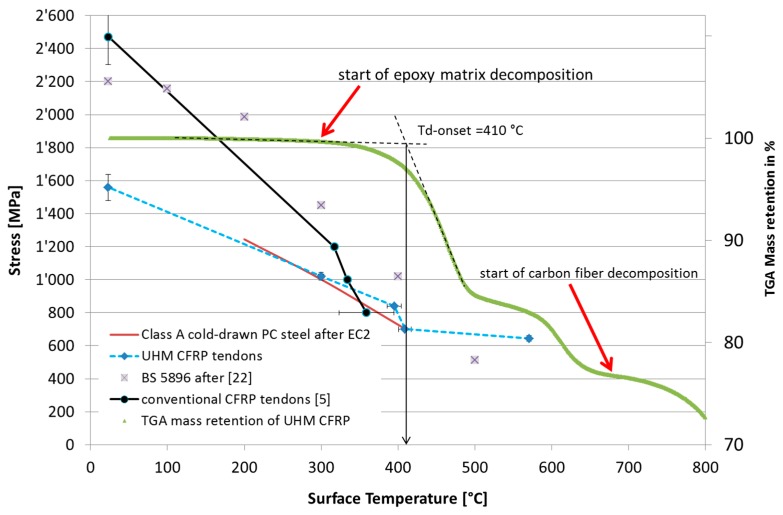
High-temperature tensile strength test results for novel UHM CFRP reinforcing tendons, compared against conventional CFRP tendons [6], BS 5896 steel prestressing wires [24], and guidance from Eurocode 1992-1-2 [25]; also including TGA mass loss curve for the novel UHM CFRP.

A comparison of the tensile strength of the novel UHM tendons with the thermal behaviour of standard (PAN-based) CFRP tendons [6] versus that obtained from prior tests on deformed high tensile steel prestressing wires of type BS 5896 [24] is shown in Figure 10. The Eurocode 1992-1-2 [25] high-temperature design tensile strength reduction curve for Class A cold drawn prestressing steel wires is also included in Figure 10 for comparison. The secondary axis of Figure 10 also shows the retained mass of the novel CFRP material during thermogravimetric analysis. Highlighted are the start of the epoxy matrix decomposition, at approximately 300 °C, and the start of the carbon fibre oxidation, at around 680 °C.

The first observation arising from Figure 10 is that typical steel prestressing wires also suffer considerable reductions in tensile strength at elevated temperatures. The figure also confirms the expectation that the Eurocode 1992-1-2 [25] tensile strength reduction recommendations are somewhat conservative (by approximately 60 to 100 °C based on the limited results presented herein) with respect to the testing methods and modern steel prestressing materials used, for example, in [24]. This was previously verified by others [6].

Figure 10 shows that both UHM and conventional CFRP tendons [6] also experience considerable reductions in tensile strength under exposure to elevated temperatures. On average, the PAN-based normal modulus CFRP tendons described in [1,6] are more sensitive to elevated temperature than a BS 5896 steel prestressing wire [24]; for example experiencing failure at a temperature about 70 °C lower than the steel at the a stress level of 1000 MPa. However, the main objective in the current study was the characterization of the elevated-temperature strength behaviour of the novel UHM CFRP tendons, which—If considered relative to their design tensile strength—Showed a better normalized retention of high temperature performance than the conventional PAN-based CFRP tendons. The design tensile strength of the normal modulus CFRP tendons is 2000 MPa [1], while that of the new UHM tendons is only 1400 MPa. The novel UHM CFRP tendons show a failure temperature of 409 °C at a sustained stress level of 50% of their design tensile strength (700 MPa), while the conventional CFRP tendons fail at an average temperature of 334 °C when loaded at 50% of their design tensile strength (i.e., 1000 MPa ) [6]. For a sustained stress level of 1000 MPa, both tendon types fail in the same temperature range between 300 and 334 °C.

Considering the shallower gradient of the loss of tensile strength of the novel UHM tendons with temperature, at 300 °C the average tensile strength of the tendons is 1021 MPa (73% of their design DTS). At 570 °C, the epoxy matrix of the tendons is almost fully decomposed, and therefore incapable of transferring shear stresses [15] amongst the individual carbon fibres (see the TGA curve in Figure 10). However, the average remaining tensile strength of the novel tendons is 644 MPa, which corresponds to 46% of DTS.

It is noteworthy that the maximum surface temperature of a CFRP tendon in an intact prestressed concrete slab of 75 mm total thickness, and subjected to an ISO 834 fire from one side, can be expected to reach between 450 and 475 °C after 90 min [8]. For this type of fully prestressed slab [1] CFRP tendon prestress is typically 40% of the tendon design DTS (i.e., 560 MPa for the UHM tendons in the current study). This is less than the tendons’ remaining tensile strength at 570 °C (644 MPa, Stdv. 18 MPa). Thus, this suggests that—at least with respect to the high temperature tensile strength performance—there is the potential to use the novel UHM CFRP tendons in CFRP pre-tensioned concrete elements for building applications [1,6] with up to 90 min of fire resistance.

The other important ‘fire-performance’ related issue that cannot be overlooked in this context is the understanding of the bond behaviour at high temperatures between the novel sand-coated UHM tendons and the concrete. For such prestressed concrete elements, CFRP anchorage regions with a limited temperature increase are essential. Combined experimental and numerical studies on these issues are being undertaken via a collaborative project between The University of Edinburgh and Empa.

It is widely considered that the upper temperature limit for CFRP used in civil engineering structural applications is given by the *T_g_* of the polymer resin matrix used in the CFRP composite profile fabrication [26]. For FRP composites used in infrastructure applications, *T_g_* is commonly measured in DMTA experiments and determined using a *T_g_*-onset definition. This definition is shown in Figure 9, where the retained storage modulus *E’*(*T*) of the specimen is plotted versus the temperature for the CFRP tendons used in the current study. The *T_g_*-onset value for the CFRP tendons was 115 to 117 °C (Table 3). For comparison, the *T_g_* value for the novel UHM tendons was also determined with a tan δ peak-definition (also shown in Figure 9), which gave—As anticipated in [27]—Somewhat higher values of 139–142 °C (Table 3). While it is clear that the loss in matrix modulus (and strength) for temperatures over *T_g_*-onset influences the decrease of the remaining tensile strength of the CFRP tendon with temperature, this reduction is limited to approximately 540 MPa in the temperature range 115 to 300 °C (Figure 10). This suggests that a considerable portion of the short-term tensile strength of the novel UHM CFRP tendons can be used at temperatures well above *T_g_* of the tendons’ epoxy matrix (if the anchorage zones of the tendons are not subjected to high temperatures). This is a potentially important finding when designing for fire resistance of a UHM CFRP reinforced or prestressed concrete structural element.

Thermogravimetric analysis in air was used to determine if the temperature of onset of resin decomposition (e.g., approximated with a tangent intersection as *T_d_*-onset in Figure 10), could be used to provide an indication of the temperatures which might lead to a considerably reduced transient thermal tensile strength of the UHM CFRP tendons. The thermal degradation was correlated and is displayed as the normalized reduction of the specimen’s mass [28] in Figure 10. It can be observed that the thermal degradation of the epoxy resin starts at approximately 300 °C, and ends at approximately 680 °C, while the oxidation of the carbon fibres begins at about 680 °C and continues until the test finishes at 900 °C. The comparison of the estimated *T_d_*-onset value of 410 °C versus the temperatures needed to cause tensile rupture suggests that *T_d_*-onset does not provide a particular indication of a necessarily ‘critical’ temperature for the tendons when stressed to reasonable service levels. Interestingly, at temperatures higher than *T_d_*-onset the reduction in transient thermal tensile strength is limited. This suggests that at such high temperatures the carbon fibre bundle (tendon) does not experience any significant stress transfer between the fibres via the decomposing epoxy matrix. The composite action is therefore lost and the ‘dry’ fibres maintain the tensile strength in the range of 640 to 700 MPa. Clearly, this only holds for the stress levels and testing procedures used in the current study, and additional research on a variety of CFRP materials and sustained stress levels is necessary for verification and corroboration.

## 6. Summary and Conclusions

A novel ultra-high modulus CFRP prestressing tendon made from coal tar pitch-based carbon fibres was characterized in terms of high-temperature tensile strength (up to 570 °C) with a series of transient thermal and steady temperature tensile tests. Digital image correlation was used to capture the high-temperature strain development during thermal and mechanical loading. Complementary thermogravimetric (TGA) and differential mechanical thermal (DTMA) experiments were also performed on the tendons to elucidate their high temperature thermal and mechanical behaviour.

The high-temperature tensile strengths determined for the novel UHM CFRP tendons are considered to be outstanding when compared to the high temperature performance of FRP reinforcements for concrete structures or conventional steel prestressing wires. A literature review in [7] revealed that at temperatures between 250 and 400 °C, most carbon/epoxy CFRP composites lose about half of their original tensile strength. The novel UHM CFRP tendons investigated in the present study have a design tensile strength (DTS) of 1400 MPa; their failure temperature at a prestress of 50% of the design tensile strength is 409 °C. Conventional PAN-based CFRP tendons investigated by the same authors [6] failed at an average temperature of only 334 °C when loaded to 50% of their design tensile strength. It is noteworthy that at 300 °C the average tensile strength of the novel UHM tendons studied was still 1021 MPa (73% of their design tensile strength). This shows that a considerable part of the short-term tensile strength of the novel UHM CFRP tendons can be used at temperatures well above the *T_g_* of the tendons’ epoxy matrix (115 °C). At 570 °C, the epoxy matrix of the tendons was almost fully decomposed and therefore incapable of transferring shear loads amongst the individual carbon fibres. This was confirmed using TGA experiments. However, the average remaining tensile strength of the novel tendons was 644 MPa, which corresponds to 46% of their design tensile strength. This relatively high remaining tensile strength at 570 °C shows that there is potential to use the novel UHM CFRP tendons in CFRP pre-tensioned concrete elements for building applications that fulfill the fire resistance criteria (e.g., for an ISO 834 design fire scenario) [1,6,8]. Additional research is needed to confirm this conclusion.

## Figures and Tables

**Table 1:** Comparison of the tensile properties of UHM CFRP tendons versus standard CFRP tendons.

Material	Fibre volume content (%)	*E*_11 calc._ [14] (GPa)	*E*_11 exp._ (GPa)	σ_11 max calc._ [14] (MPa)	σ_11 max exp._ (MPa)	σ_11 failure exp._ (%)
UHM CFRP tendon K13916	66	502	509	2112	1561	0.30
Standard CFRP tendon UTS5631 [1]	65	150	156	3096	2471	1.54

**Table 2:** Results of transient thermal tensile tests of prestressed UHM tendons. n.a. means not applicable.

Constant stress (MPa) (% of DTS)	Mean failure temperature (°C) (Stdv)	CFRP strain at failure estimated by DIC (%)
560 (40%)	No failure at 570 °C	-
700 (50%)	409 (8.3)	0.18%–0.22%
840 (60%)	395 (9.0)	n.a.

**Table 3:** *T_g_* values for UHM CFRP tendons measured during first and second heating runs of DMTA testing.

Sample/Run	*T_g_* (onset E’) (°C)	*T_g_* (max tan δ) (°C)
**1/1**	116 ± 1	142 ± 1
**1/2**	115 ± 1	139 ± 1
**2/1**	117 ± 1	140 ± 1
**2/2**	115 ± 1	139 ± 1
